# Genetics Abnormalities with Clinical Impact in Primary Cutaneous Lymphomas

**DOI:** 10.3390/cancers14204972

**Published:** 2022-10-11

**Authors:** Fernando Gallardo, Ramon M. Pujol

**Affiliations:** 1 Department of Dermatology, Hospital del Mar, Parc de Salut Mar, Passeig Maritim 25-29, 08003 Barcelona, Spain; 2 Department of Medicine and Life Sciences, Universitat Pompeu Fabra, 08003 Barcelona, Spain; 3 Department of Medicine, Universitat Autònoma de Barcelona, 08003 Barcelona, Spain

**Keywords:** cutaneous lymphoma, genetics

## Abstract

**Simple Summary:**

The genetic landscape of cutaneous T-cell lymphomas analyzed by sequencing high throughput techniques shows a heterogeneous somatic mutational profile and genomic copy number variations in the TCR signaling effectors, the NF-κB elements, DNA damage/repair elements, JAK/STAT pathway elements and epigenetic modifiers. A mutational and genomic stratification of these patients provides new opportunities for the development or repurposing of (personalized) therapeutic strategies. The genetic heterogeneity in cutaneous B-cell lymphoma parallels with the specific subtype. Damaging mutations in primary cutaneous diffuse large B-cell lymphoma of the leg type, involving MYD88 gene, or BCL6 and MYC translocations or CDKN2A deletions are useful for diagnostic purposes. The more indolent forms, as the primary cutaneous lymphoma of follicle center cell (somatic mutations in TNFRSF14 and 1p36 deletions) and the cutaneous lymphoproliferative disorder of the marginal zone cells (FAS gene), present with a more restricted pattern of genetic alterations.

**Abstract:**

Primary cutaneous lymphomas comprise a heterogeneous group of extranodal non-Hodgkin lymphomas (NHL) that arise from skin resident lymphoid cells and are manifested by specific lymphomatous cutaneous lesions with no evidence of extracutaneous disease at the time of diagnosis. They may originate from mature T-lymphocytes (70% of all cases), mature B-lymphocytes (25–30%) or, rarely, NK cells. Cutaneous T-cell lymphomas (CTCL) comprise a heterogeneous group of T-cell malignancies including Mycosis Fungoides (MF) the most frequent subtype, accounting for approximately half of CTCL, and Sézary syndrome (SS), which is an erythrodermic and leukemic subtype characterized by significant blood involvement. The mutational landscape of MF and SS by NGS include recurrent genomic alterations in the TCR signaling effectors (i.e., PLCG1), the NF-κB elements (i.e., CARD11), DNA damage/repair elements (TP53 or ATM), JAK/STAT pathway elements or epigenetic modifiers (DNMT3). Genomic copy number variations appeared to be more prevalent than somatic mutations. Other CTCL subtypes such as primary cutaneous anaplastic large cell lymphoma also harbor genetic alterations of the JAK/STAT pathway in up to 50% of cases. Recently, primary cutaneous aggressive epidermotropic T-cell lymphoma, a rare fatal subtype, was found to contain a specific profile of JAK2 rearrangements. Other aggressive cytotoxic CTCL (primary cutaneous γδ T-cell lymphomas) also show genetic alterations in the JAK/STAT pathway in a large proportion of patients. Thus, CTCL patients have a heterogeneous genetic/transcriptional and epigenetic background, and there is no uniform treatment for these patients. In this scenario, a pathway-based personalized management is required. Cutaneous B-cell lymphoma (CBCL) subtypes present a variable genetic profile. The genetic heterogeneity parallels the multiple types of specialized B-cells and their specific tissue distribution. Particularly, many recurrent hotspot and damaging mutations in primary cutaneous diffuse large B-cell lymphoma of the leg type, involving MYD88 gene, or BCL6 and MYC translocations and BLIMP1 or CDKN2A deletions are useful for diagnostic and prognostic purposes for this aggressive subtype from other indolent CBCL forms.

## 1. Introduction

Primary cutaneous lymphomas (CL) comprise a heterogeneous group of T and B-cell non-Hodgkin lymphomas (NHL) that arise from skin resident lymphoid cells and may extend to the lymph nodes, peripheral blood, and eventually extranodal sites [1]. The different clinical variants of CL have been recognized in the recent WHO classification of hematopoietic and lymphoid tissue neoplasms [1,2]

Primary cutaneous T-cell lymphomas (CTCL) are the most important group, accounting for 70% of CL, and have an estimated incidence of up to 10 new cases per million people per year [1,3]. Mycosis Fungoides (MF) and Sézary syndrome (SS) are the most representative entities and show a heterogeneous mutational landscape that comprises elements of the TCR signaling, NF-κB and the DNA damage/repair pathways, being copy number variations (CNV) more common than somatic mutations in the JAK/STAT pathway. Primary cutaneous anaplastic large cell lymphomas (pcALCL) have rearrangements at the IRF4/DUSP22 locus and common genetic alterations of the JAK/STAT pathway. Recently, recurrent JAK2 rearrangements have been reported in primary cutaneous CD8+ aggressive epidermotropic cytotoxic T-cell lymphoma (pcAETCL), a rare lethal subtype of CTCL, and may represent a new targeted therapeutic approach. In addition, other aggressive cytotoxic CTCL (primary cutaneous γδ T-cell lymphoma [pcGDTL]) show mutations in the JAK/STAT pathways in a large proportion of patients [1,2,4].

There is no curative treatment for MF, SS and other CTCL, and there is an urgent need for new effective well-tolerated treatments with a safe profile and that maintain long-term activity. In recent years the knowledge of specific therapeutic targets has allowed for the design of immunotherapies (both cytotoxic and immunoregulatory) such as brentuximab, mogamulizumab, or check-point inhibitors (PD1/PDL1 inhibitors), which have modified the disease-free survival of these patients in the short term. Given the heterogeneity of these CTCL tumours, it is essential to obtain personalized therapies (precision medicine) adapted to the different mechanisms and subtypes of CTCL and the characteristics of each patient in particular. The identification of new associated biomarkers will be relevant for the stratification of patients and in defining which ones will benefit from onco-specific drugs or pathway inhibitors. It can also be useful to establish prognostic value markers that allow for the adapting of the therapeutic attitude [4].

Primary cutaneous B-cell lymphomas (CBCL) form a present a variable genetic profile according its specialized B-cells and their specific tissue distribution. Primary cutaneous lymphoma of the follicle center cells presents common somatic mutations in TNFRSF14 and CREBBP or associates 1p36 deletions, but at lower frequencies than in systemic follicular lymphomas. Genetic alterations in the FAS gene have been described in a high percentage of cases and, although other translocations specific to extranodal (non-cutaneous) marginal zone lymphomas of the MALT (mucosa-associated lymphoid tissue) type can be observed, such as t(1;14)(p22;q32), t(11;18)(q21;q21), or t(14;18)(q32;q21)-IgH/MALT, the number of cases is very limited and they are of little diagnostic value. Primary cutaneous diffuse large B-cell lymphoma of the leg type involves characteristic mutations in the MYD88 gene or CDKN2A deletions [1,2].

Many of these genomic alterations in CL, being present in other mature NHL, may represent opportunities for the development of monitoring and therapeutic strategies that are currently in use or under investigation in other hematological malignancies.

## 2. Primary Cutaneous T-Cell Lymphomas: Mycosis Fungoides and Sézary Syndrome

MF accounts for 50% of all CL and is clinically manifested by a slow progression of the disease over years or decades from cutaneous macules to infiltrated plaques, until patients develop skin tumors or lymph node involvement, which significantly worsens the prognosis. SS is an aggressive disease that almost exclusively affects adults or elderly patients (mainly males) and is classically manifested by pruritic erythroderma, generalized lymphadenopathy, and atypical circulating of large mononuclear cells with convoluted nuclei (Sézary cells). The overall survival of SS is similar to that of advanced-stage MF, approximately30% at 5 years [5,6,7].

MF/SS are generally malignant neoplasms of CD4+ T cells that exhibit a T-helper memory phenotype (CD45RO). The clonal expansion of malignant T cells in advanced stages occurs together with a loss or restriction of the diversity of the repertoire of T cell receptors (TCR), an increased activity or regulatory function by the same malignant cells, and a decrease in the number of CD8+ T cells. Malignant SS T cells co-express circulating receptor molecules such as CCR7, CD62L, L-selectin, and the central memory T cell marker CD27. In contrast, MF cells present a skin-resident effector memory T cell phenotype lacking CCR7/L-selectin, CD103, or CD27. They express CCR4, CD69, and the cutaneous lymphocyte antigen (CLA). Whole exome sequencing (WES) studies suggest that malignant clonotypes in MF develop in T-cell progenitors prior to TCRβ or TCRα rearrangements [8,9,10].

### 2.1. Genetic and Epigenetics Abnormalitites in MF/SS

Next-generation sequencing (NGS) approaches have been successfully applied, looking at the MF/SS mutational landscape and have identified putative genomic alterations and driver mutations in genes involved in the development and progression of these diseases. A significant proportion of studies have been focused on SS since homogeneous sampling is easier to analyze under NGS platforms. MF and SS harbor complex and heterogeneous (non disease-specific) genetic and epigenetic alterations. Many of the identified driver genes are shared by both diseases, and it remains open to discussion whether they represent extremes of the same spectrum of diseases or are different disorders [11,12,13,14,15,16,17,18,19,20,21,22,23,24,25]. Broad similarities across disease stages have been observed, although more structural variants have been detected in leukemic disease, leading to highly recurrent deletions of putative tumor suppressors that are uncommon in early-stage skin-centered MF (i.e., TP53 gene). Fusion genes play an important role in tumor development because they might result in disruption of either tumor suppressor genes or the activation of proto-oncogenes [11,25,26,27,28]. C>T transitions represent the main mechanism of mutations and the possibility of a contribution of UV exposure to MF/SS has been discussed [13,14,26]. On the other hand, alkylating-related signatures seem to be restricted to early MFs, and age-related signatures are enriched, but not exclusively, in SS. Many driver mutations are present in both forms of the mutations but are more prevalent in late-MF and in SS compared with skin-limited CTCL [26,27]. Different alterations on T-cell activation and NF-κB signaling, apoptosis, chromatin remodeling, DNA damage response together with signaling pathways including JAK/Signal Transducer and Activator of Transcription (STAT) and cell-cycle checkpoints have been most commonly detected [26,28].

#### 2.1.1. Spectrum of Somatic Point Mutations

Recurrent mutations in the DNA damage/repair elements TP53 (18%) and splice-site mutation affecting the FAT (FRAP, TRAPP) domain of ATM are commonly detected [19,26]. The TCR/NF-κB signaling effectors carrying somatic mutations include PLCG1 (9–21%), TNFRSF1B or CARD11 (5–7%) and GLI3, which may interact with BCL10, a positive regulator of cell apoptosis and NF-κB activation [11,13,16,17,26]. Interesting hotspot point mutations in other oncogenes from this pathway such as NFKB1 (p.H67Y), KLF2 (p.H346Q/N/Y), and JUNB (p.A282V) and damaging mutations in the tumor suppressor genes FUBP1 and ANO6 have been newly identified in a large number of samples from diverse MF/SS stages [26,29]. Other interesting recurrent genes affected by somatic point mutations (but with a low prevalence of less than 5%) include stop-gain mutations in ITPR1/2 (a functional partner of the anti-apoptotic BCL2 gene), DSC1 and PKHD1L1 (cellular immunity) genes [11]. Singleton somatic mutations in RIPK2 and IL6 (CD4+ cells activation, TCRα/β differentiation), missense mutation in the (recombination activity) RAG2 gene and mutations in STAT5B gene have been reported in CTCL [12,13]. Interestingly, the status of PD1 mutations vs deletions seems to predict the patient survival in CTCL. Neoplastic T-cells in SS frequently express PD-1, and although gene mutations in CTCL commonly promote TCR-dependent proliferation, most CTCL cases show the “T-cell exhaustion” phenotype (PD1, TGT1 and CXCL13-positive) and could not proliferate after TCR stimulation. However, cases showing loss of PD-1 (deletion) may reverse this phenotype, increasing cell proliferation and prompting a worse clinical course [13,16,26]. Table 1 summarizes many of these genetic abnormalities reported in MF/SS.

As many of these genetic abnormalities are shared by other different hematological neoplasms, they may represent clear candidates for genetic screening panel designs of T-cell NHL to improve diagnosis or monitoring (Table 2) [30].

#### 2.1.2. Genomic Copy Number Variants

Former approaches using comparative genomic hybridization array techniques detected recurrent large genomic and chromosome imbalances in tumor stage MF and SS that have now been confirmed by NGS. CNV particularly involve the 17p, 9p21 and 10q deletions and 17q amplification [31,32].

The landscape of somatic duplications in CTCL includes large chromosome bands in 8p23.3–q24.3 and 17p11.2–q23.2, 10p15.3–p12.2, and several focal somatic duplications that may encompass genes that play a role in tumorigenesis (ANKRD26, BCL7C, CRIP3, RAMP3 or TRBG4) [11,12,13,14,16,18,19,26].

On the other hand, somatic deletions at 10p11.1–q26.3, 11q23.3–q25, 19p13.2–p13.3, or 17p12–p13.3 encompass several interesting genes, such as loss of the tumor suppressor TP53 or DAD1 (pro-apoptosis). Genomic gains of RASA2, a mitogenic-activated protein kinase (MAPK) signaling pathway proto-oncogene or CBLB (proto-oncogene that activates T cells by inhibiting PLCG1) are very interesting candidates for further studies [11,19].

In addition, genetic CNV gains in DNMT3A, ARID1A, CTCF, NCOR1, KDM6A, SMARCB1, ZEB1, PRKCB, PTPRN2, and RLTPR are more frequent than somatic mutations, and occur in TNFAIP3, CSNK1A1 [12,13,18,24,25], and in various elements of the JAK/STAT pathway (STAT5B, STAT3) [11,12,13,14,22,24,26]. Deletions in GRAP (TCR signaling), AGAP6, ZBTB7A, and SBNO2 (cytokine signaling) also point to possible candidate genes in the pathogenesis of MF/SS [26,27]. The most prevalent and relevant CNVs are represented in Table 1.

#### 2.1.3. Complex Chromosomal Rearrangements

Complex chromosomal rearrangements or fusion events are rare and also highly heterogeneous (TYK2-UPF1, COL25A1-NFKB2, FASN-SGMS1, SGMS1-ZEB1, SPATA21-RASA2, PITRM1-HK1, or BCR-NDUFAF6, among others) but they represent interesting candidates as potential biomarkers or therapeutic targets in CTCL. FASN (fatty acid protein synthase) together with SGMS1 (sphingomyelin synthase 1) are involved in several types of cancer and are regulated by the ABL proto-oncogene. ZEB1 encodes a zinc finger transcription factor and acts as a transcriptional repressor of IL2 (T-cell differentiation) [12,13,14,17]. TYK2 is essential for the differentiation and function of different immune cells. NFKB1, KLF2 or NFKB2 as well as other translocations that affect genes of the T cell differentiation pathway (TCR/NF-κB) have been repeatedly implicated in CTCL pathogenesis [11,13,16]. The elevated level of hexokinase 1 (PITRM1-HK1 fusion event) causes tumor cells to avoid apoptosis; the fusion of BCR-NDUFAF6 (ABL) is amenable to targeting in patients with SS as well as that of CTLA4 and CD28 fusion [16,33].

#### 2.1.4. Epigenetics in CTCL

Some mutated genes in CTCL are epigenetic modifiers. The methylation of cytosine residues to 5-methocytosine is mediated by DNA methyltransferases (DNMTs), so gain-of-function mutations and CNVs of DNMT3A represent an interesting mechanism of genomic/epigenomic cooperation that can explain many changes in functions of many genes in CTCL. Furthermore, they have been identified with a high frequency in MF/SS, as occurs in many other hematological malignancies, which increases their relevance. Other alterations in epigenetic regulatory genes are the loss of DNA (hydroxy-) methylation mediated by the family of translocations ten-eleven (TET), mutations in isocitrate dehydrogenases, which inhibit TET proteins, and ARID1A/B (which form part of the chromatin patterning complexes) and the MLL genes, which mediate histone methyltransferases [12,26].

Epigenetic abnormalities complement the genomic landscape of MF/SS. Whole genome DNA methylation status has been scarcely deciphered in SS studies. Methylation status of CMTM2, C2orf40, G0S2, HSPB6, PROM1, o PAM genes have been identified as potential diagnostic epigenetic markers to differentiate SS from inflammatory erythrodermas [34].

The tumor suppressor genes CDKN2A and CDKN2B, which encode the cell cycle proteins p16, p14ARF and p15, are located in the 9p21.3 region, and are frequently lost in MF in tumor stages or in transformation, and are associated with a poor prognosis [35,36]. The genetic loss in this locus can be homozygous but often is associated with promoter hypermethylation of the other allele [37].

Differentially expressed microRNAs have been identified between inflammatory processes and/or normal skin and in MF in early stages. Thus, it has been observed that the microRNAs, miR-155, miR-146a, 146b-5p, miR-342-3p and let-7i were overexpressed, and the microRNAs, miR-203 and miR-205 decreased in MF. The group of microRNAs, miR-NAs 26a, miR-222, miR-181a and miR-146a, likewise, are differentially expressed between tumor and inflammatory cases. Table 3 brings together the main microRNAs differentially expressed in the different forms of CTCL [38,39].

#### 2.1.5. Current and Potential Therapeutic Implications: Towards a Personalized Medicine in CTCL

CTCL are malignant neoplasms that are genetically heterogeneous even within each clinical subtype, so a single therapy may not be suitable for all patients. Determining the specific profile of the specific mutations of each patient or subtype of CTCL is clearly a challenge with direct implications in the diagnosis, monitoring and design of (personalized) therapies. With knowledge of the (heterogeneous) genetics of CTCL and the stratification of patients by altered signaling pathways, different targeted therapeutic approaches can be proposed and are illustrated in Table 4. Therapies targeting the PI3K pathway (duvelisib), NF-κB inhibitors (bortezomib), alisertib (oral Aurora A kinase inhibitor), or JAK/STAT inhibitors, widely used in inflammatory conditions or hematologic malignancies, may show promise in CTCL and are awaiting efficacy and tolerance results from clinical trials. The presence of gene alterations related to immune evasion that lead to abnormal expression of PD1, PD-L1 and PD-L2 and co-stimulatory elements, such as CD28-ICOS, have prompted research on the use of anti-PD1/PD-L1 antibodies [22]. Finally, unraveling the epigenetics in CTCL may also have therapeutic implications. Thus, histone deacetylase (HDAC) inhibitors, such as vorinostat or romidepsin, are already approved in the US for use in CTCL, and resminostat is in clinical trials in Europe. In addition, cobomarsen, an oligonucleotide inhibitor of miR-155, is currently being investigated for use in CTCL [40].

## 3. Other Cutaneous T-Cell Lymphomas Distinct from Mycosis Fungoides and Sézary Syndrome

Other clinical variants of CTCL have been recognized in the WHO classification that include the indolent primary cutaneous CD30(+) lymphoproliferative disorders, extranodal natural killer T-cell lymphoma (ENKTCL), pcGDTL, pcAETCL, subcutaneous panniculitis-like T-cell lymphoma (SPTCL), and primary cutaneous T-cell lymphoma, not otherwise specified (pcPTCL-NOS) [1,2].

### 3.1. Cutaneous CD30(+) Lymphoproliferative Disorders

The group of CD30(+) cutaneous lymphoproliferative disorders represents a spectrum of processes ranging from lymphomatoid papulosis (LyP), characterized by the presence of spontaneously regressing papules, to pcALCL, presenting as single or multiple cutaneous tumors with a low propensity to spread. Cases of pcALCL lack the common genetic alterations found in systemic CD30(+) ALCL, but up to 20% of pcALCL have rearrangements at the IRF4/DUSP22 locus. NGS technology detected mutations that affect the IL6-JAK1-STAT3 pathway in approximately 15–30% of cases, which places JAK/STAT as a candidate target for new personalized treatments in this CTCL subtype. Other genetic alterations include mutations in DNMT3A and TP53, and the PI3K or MAPK pathways. Other recurrent events affecting cancer-associated genes include the deletion of PRDM1 or TNFRSF14, the gain of EZH2 and TNFRSF8, mutations in LRP1B, PDPK1, and PIK3R1, and rearrangements of GPS2, LINC-PINT, or TNK1 [41,42,43,44,45]. Additionally, for pcALCL, array-CGH analyses have revealed chromosomal imbalances in CTSB (8p22), RAF1 (3p25), REL (2p12) and JUNB (19p13.2) and allelic deletion at 9p21–22, causing inactivation of CDKN2A tumor suppressor gene [46].Therefore, inhibition of these proliferation-promoting pathways should also be explored as potential alternative therapies.

Clinical behavior, phenotypical and genetic profile allow differentiation of pcALCL from systemic CD30(+) ALCL. Systemic CD30+/ALK+ ALCL has ALK gene rearrangements, and the combined nuclear and cytoplasmic expression of ALK protein strongly suggests an underlying t(2;5) translocation of ALK with nucleophosmin. The cytoplasmic expression of the ALK protein is associated with other fusion partners of the ALK gene such as TRAF1, ATIC or TPM3. Systemic CD30+/ALK− ALCL lack an ALK gene rearrangement and subsequently any ALK expression, but has other alterations, such as 6p25 rearrangements involving the IRF4/DUSP22 locus (the same as pcALCL which are negative for ALK expression, as well). Cases with IRF4/DUSP22 rearrangement have a more favorable clinical course than those systemic CD30+/ALK− ALCL which do not carry the IRF4/DUSP22 rearrangement, and similar to CD30+/ALK+ ALCL or pcALCL. On the other hand, patients with systemic CD30+/ALK− ALCL with TP63 rearrangements have a poor prognosis, while the absence of ALK, DUSP22, and TP63 rearrangements (triple negative) results in an intermediate prognosis. Finally, ALCL associated with breast implants is a rare subset of CD30+ALK− ALCL with an overall favorable prognosis. Deletions on chromosome 20q13.13 have been identified in two-thirds of cases of this latter subtype of ALCL [47]. The molecular alteration at the IRF4/DUSP22 locus is less frequent in LyP than in pcALCL and accounts for fewer than 5% of cases [48]. LyP may be associated withother T-cell lymphomas, particularly MF and pcALCL, being clonally related, which suggests that a non-random genetic event initiates the disease [49]. The management of LyP is that of an indolent CL, based on its excellent prognosis; however, this good prognosis is altered if LyP is associated with other lymphomas (up to 40% of cases in some series) [50,51]. The main genetic alterations reported in cutaneous and systemic ALCL are represented in Table 5.

### 3.2. Subcutaneous Panniculitis-like T-Cell Lymphoma

SPTCL is a rare primary cutaneous lymphoma composed of αβ CD8 cytotoxic T cells with a much less aggressive course than pcGDTL, although 15–20% of cases can develop hemophagocytic syndrome (HPS) and a fatal course. In approximately 20% of cases, the concomitant association or an overlapping presentation with lupus panniculitis has been described. Loss-of-function mutations in the HAVCR2 gene (encoding T-cell immunoglobulin mucin 3 [TIM-3]) have been described in up to 60–80% of cases, either as somatic or germline mutations, as a factor that predisposes to SPTCL development. Mutations in HAVCR2 alter highly conserved residues of TIM-3, an immune response modulator and, consequently, giving rise to an uncontrolled activation of the immune system. In patients of East Asian and Polynesian background, the pathogenic variant c.245A>G (HAVCR2Y82C) is usually detected, whereas for patients of European background, the c.291A>G (HAVCR2T101I) variant is usually demonstrated. Patients with mutated HAVCR2 (HAVCR2Y82C) seemed to be associated with younger age, the development of HPS or hemophagocytic lymphohistiocytosis-like systemic illness, and short relapse-free survival. Mutations in UNC13D, PIAS3, and KMT2D, and the upregulation of CCR4 were more frequent in non-mutated HAVCR2 SPTCL, and enrichment in genes involving IL6-JAK-STAT3 signaling and TNF-α signaling via NF-κB have also been reported [52,53].

### 3.3. Primary Cutaneous γδ T-Cell Lymphoma

The pcGDTL is a poor prognosis neoplasm with a 5-year overall survival of 10% which often shows systemic symptoms with features of HPS. NGS and TCR sequencing indicated that the cells-of-origin of pcGDTL are Vδ1 cells predominant in the epidermimotropic and dermal variants and Vδ2 cells in the panniculitic counterpart. Vδ1 and Vδ2 lymphomas show similar targetable mutations more commonly affecting the JAK/STAT, MAPK, MYC, and chromatin editing pathways [54].

### 3.4. Primary Cutaneous CD8(+) Aggressive Epidermotropic Cytotoxic T-Cell Lymphoma

Primary cutaneous CD8(+) aggressive epidermotropic lymphoma pcAETCL is a rare fatal subtype of T-cell NHL presenting complex karyotype and clonal evolution that reflects genomic instability. Gains of 7q, 8q24.3, 17q and losses of 9p21.3 (CDKN2A-CDKN2B) and 17p including the TP53 gene have been reported;however, very recently, the pcAETCL carries JAK2 gene fusions that may render them especially susceptible to JAK inhibitors [55,56,57,58].

### 3.5. Extranodal NK/T-Cells Lymphoma, Nasal Type

ENKTCL is an aggressive lymphoma associated with Epstein-Barr virus (EBV) infection that typically but not exclusively presents in the nasal and paranasal areas. It develops in adult patients from Caribbean or East Asian countries. The genetic profile of ENKTCL is characterized by mutations in JAK/STAT components, tumor suppressor genes (TP53 or MGA), epigenetic modifiers (KMT2D, ARID1A), and BCOR corepressor loss-of-function mutations. Gains in 8q24 [MYC], 2q gain and losses on chromosomes 6q16–q27, 6q21, or 11q22–q23 have also been described in ENKTCL [59]. Hydroa vacciniforme lymphoproliferative disease, formerly referred to as hydroa vacciniforme-like, encompasses a EBV-associated process that may harbor driver mutations in STAT3 and KMT2D among others genes, similar to ENKTCL, and may also represent future targets for therapy, as well [60].

### 3.6. Primary Cutaneous T-Cell Lymphoma, Not Otherwise Specified 

The pcPTCL-NOS are a heterogeneous group of CTCL showing variable clinical (often aggressive) and immunohistochemical features giving rise to some difficulties with regard to classification among the CTCL variants, and may even exhibit T-cell helper follicular (THF) markers such as PD-1, CD10, CXCL13, BCL6, and ICOS and share pathologic characteristics with angioimmunoblastic T-cell and THF lymphomas [61]. Similarly to other non-cutaneous recurrent mutations that have been described in cases of PTCL-NOS affecting epigenetic regulators (KMT2C and ARID1A) and in TCR signaling molecules (PLCG1 and CD28). Mutations of TET2, RHOA (which induces THF lineage specification and promotes T-cell lymphomas in cooperation with loss of TET2), DNMT3A, and IDH2 are also detected. Non-THF, PTCL-NOS subtypes carry mutations and deletions of TP53 and/or CDKN2A. The identification of some cases with mTOR mutations raises the benefit of targeted therapy [62,63].

## 4. Primary Cutaneous B-Cell Lymphomas

Primary cutaneous B-cell lymphomas (CBCL) represent approximately 30% of the primary cutaneous lymphomas. They include a heterogeneous group of entities with different clinicopathological and evolutionary characteristics. They usually present as papules, nodules or tumors, solitary or multiple, occasionally appearing grouped or as multifocal generalized lesions. Three well-defined groups can be distinguished: primary cutaneous lymphoma of follicle center cells (pcBCLf), primary cutaneous lymphoma of marginal zone cells (pcBCLm), now proposed under the terminology of cutaneous lymphoproliferative disorder of the marginal zone cells, and primary cutaneous diffuse large B-cell lymphoma of the leg type (pcDLBCL). Intravascular large B-cell lymphoma (IVLBCL) and several provisional entities are also included in the WHO/EORTC classification [1,2].

pcBCLm and pcBCLf are lymphoproliferative processes with indolent clinical behavior and are usually manifested as non-ulcerated stable plaques or nodules while pcDLBCL of the leg type is more frequent in older individuals, appearing as fast-growing plaques or nodules with a tendency to ulceration, and represents a process of aggressive evolution. The therapeutic approach of pcDLBCL is similar to that of a systemic DLBCL [64].

CBCL are characterized histopathologically by nodular (pcBCLm and pcBCLf) or diffuse dermal infiltrates (pcDLBCL), occasionally extending into subcutaneous tissue without epidermal involvement and sparing a superficial band of the papillary dermis. Occasionally, expanded large lymphoid follicles (in pcBCLf) or residual follicles colonized by the underlying lymphoid proliferation (in pcBCLm) can be observed. The morphological characteristics of the infiltrating cells are variable depending on the CBCL subtype, either small or medium sized cells (in pcBCLm and pcBCLf) or large cells and immunoblasts (in pcDLBCL, leg type). Neoplastic cells usually express mature B-cell antigens and the diagnostic procedure includes the demonstration of a monoclonal rearrangement of the immunoglobulin heavy or light chain genes or the restriction of immunoglobulin light chain expression by immunohistochemical or in situ hybridization techniques [1,2,64].

### 4.1. Primary Cutaneous FollicularB-Cell Lymphoma 

Neoplastic cells in pcBCLf correspond to mature B lymphocytes (CD19+, CD20+, CD79a+, PAX-5+), which express markers of germinal center cells (BCL-6, CD10) and, in contrast to nodal centrofollicular lymphomas, are usually BCL2-. Neoplastic cells do not express activated B cell markers (MUM1 or FOXP1), which makes it possible to differentiate them from pcDLCBL of the leg type [1,2].

A monoclonal B cell proliferation is detected in the majority of cases. Unfortunately, no specific genetic alterations have been identified. Translocation (14;18)(q32; q21) involving IgH/BCL2 genes, characteristic of systemic follicular lymphomas, is usually not detected. In rare instances, pcBCLf cases expressing BCL2 as a consequence of BCL2 gene breaks or 1p36 deletion have been reported. Occasionally, translocations between IgH and BCL6 genes, amplifications in 2p16.31-REL or deletions of 14q32.32 have also been described (see Table 6) [65]. Somatic mutations detected in pcBCLf were TNFRSF14 (40%, plus 10% with 1p36 deletions), followed by CREBBP, TNFAIP3 or KMT2D (20%). KMT2D, CREBBP, and BCL2 were significantly less commonly mutated in PCFCL than in systemic follicular lymphomas [66]. Recently, BCL2 rearrangement, chromatin-modifying gene mutations (CREBBP, KMT2D, EZH2, EP300) and the proliferation index have been proposed to classify pcBCLf specimens based on the likelihood of concurrent or future systemic spread. Since imaging may miss low-burden internal disease in some cases of systemic follicular lymphomas with cutaneous spread, many of these cases may represent systemic lymphomas misclassified as pcBCLf [67].

### 4.2. Primary Cutaneous Lymphoma of Marginal Zone Cells (Cutaneous Lymphoproliferative Disorder of the Marginal Zone Cells)

Primary cutaneous marginal zone cell lymphoma (pcBCLm) is included in the group of MALT-type lymphomas. MALT lymphomas are indolent neoplasms that develop in extranodal or mucosal locations such as the stomach, salivary glands, orbit, thyroid, breast, or lung. The pcBCLm could be considered the cutaneous variant of MALT-type extranodal lymphoma [1,2,68,69]. The term “cutaneous lymphoproliferative disorder of the marginal zone cells” has been proposed in the latest WHO classification.

These cells seem to develop in tissues where there is persistent lymphoid activation as a result of chronic antigenic stimulation (*Borrelia burgdorferi* infection, vaccines, tattoos). When the marginal zone lymphoid infiltrate becomes genetically unstable, that is, it acquires genetic alterations such as trisomy 3, trisomy 18, t(1;14)(p22;q32), t(11;18)(q21;q21), t(14;18)(q32;q21)-IgH/MALT, t(3;14)(q27;q32), t(3;14)(p14.1;q32) or FAS mutations, the lymphomatous transformation occurs. Such cytogenetic alterations observed in all MALT lymphomas are detected in a limited number of pBCLm cases (see Table 6) [70,71].

Neoplastic cells are small to medium-sized lymphocytes with a monocytoid-like appearance and a variable number of lymphocytes with plasmacytic morphology and plasma cells that are often observed in the periphery of lymphoid aggregates. Neoplastic cells show an immunophenotypic profile of mature B lymphocytes (CD20+, CD22+, CD70a+), are BCL2+ and negative for CD10 and BCL6 (follicular center cell antigens). Class-switched immunoglobulin expression IgG+/CXCR3- (expressing also IgG4) appears to be more common that non-class switch IgM+/CXCR3+ expression similar to other MALT-type lymphomas. Monotypic expression of immunoglobulin light chains can be demonstrated in a majority of cases [68].

### 4.3. Diffuse Large B-Cell Lymphoma of the Leg Type

Diffuse large B-cell lymphoma of the leg type (pcDLBCL) is a lymphoproliferative process composed of large lymphoid cells (centroblasts, immunoblasts) lacking germinal center formationand presentingan activated B cell phenotype, with the expression of BCL2, MUM1, and FOXP1 antigens. Neoplastic lymphoid cells generally do not express CD10, are occasionally BCL6+, and usually express MYC, IgM, and p63 [1,2,72].

NGS approaches have identified an original mutational landscape of pcDLBCL with a very restricted set of highly recurrent (up to 60–75%) mutations, CNV or neutral loss of heterozygosity, particularly involving MYD88 (p.L265P variant), CARD11, PIM1, and CD79B. Other genes involved in B-cell signaling, NFκB activation or DNA remodeling have also been found to be altered, notably TBL1XR1 (33%), MYC (26%), CREBBP (26%), IRF4 (21%) or HIST1H1E (41%). Amplifications or translocations in IgH, BCL6 or FOXP1 genes, genetic losses and hotspot mutations involving genes of the same pathway as well such as CDKN2A/2B, TNFAIP3/A20, PRDM1, TCF3, and CIITA provide a rationale for using selective inhibitors of the B-cell receptor to treat pcDLBCL of the leg type. The main genetic features in pcDLBCL are illustrated in Table 6. MYC and BCL2 are commonly expressed in cDLBCL of the leg type but double hits involving the MYC gene are rare and the prognostic value of MYC rearrangements seems debatable. However, mutations involving the BCR pathway (see above) may contribute to the resistance to first-line Rituximab-CHOP and deletions or hypermethylation of the p16 promoter appear to be associated with a poor prognosis as well [72,73,74].

#### Other Disorders Classified as Cutaneous Diffuse Large B-Cell Lymphomas

Intravascular large B-cell lymphoma (IVLBCL) is a rare subtype of DLBCL which often presents with characteristic cutaneous livedoid lesions andcentral nervous system involvement. Large atypical lymphoid cells are characteristically observed within the vascular lumens and corresponded to mature B lymphocytes (CD20+, CD79+) with an activated ABC phenotype MUM-1+/BCL2+/CD5+/PDL1+ and that are negative for CD10, with a high prevalence of MYD88 and CD79B mutations [75].

Several entities classified as diffuse large B-cell lymphoproliferative disorders with cutaneous presentation may represent a diagnostic challenge to differentiate from pcDLBCL. Here, the genetic/molecular profile described above takes on paramount importance in reaching the diagnosis. Among these clinical situations can be included some EBV-driven conditions that present primarily in the skin, or involving other extranodal sites such as EBV-positive mucocutaneous ulcer, lymphomatoid granulomatosis or plasmablastic lymphoma. Finally, some cases that do not fit any of the aforementioned well-characterized disorders can be considered as DLBCL not otherwise specified.

## 5. Concluding Remarks

CTCL carry a complex genomic landscape with several somatic point mutations, CNVs, and fusion events that could contribute to the pathogenesis of the disease. This genetic heterogeneity parallels the different subtypes of specialized T-cells. However, some recurrent hotspots and damaging mutations as well as CNV are shared by the different CTCL subtypes. Pathways that are repeatedly altered include the JAK/STAT, cell cycle/PI3K, TCR activation or NF-κB signaling.

Based on the evidence from different studies, it is likely that different genes such as PLCG1, CARD11, STAT5B, STAT3, FASN, NFKB1 or ZEB1, among many others, could be detected in a variable proportion of patients. Overlapping genomic features are present in multiple non-cutaneous T-cell neoplasms, also including genetic alterations in chromatin regulators such as TET, IDH or DNMT3.

The identification of recurrent alterations in CTCL will allow the design of genetic screening panels improving the diagnosis and better monitoring of the patients. In addition, a number of somatic deletions and duplications identified suggest that chromosomal instability is a feature of CTCL and should be further explored with additional genomic technology. Finally, a mutational and genomic (CNVs and fusion genes) stratification of CTCL patients provides new opportunities for the development or repurposing of therapeutic strategies to face the different entities included with the group of CTCL.

In the case of CBCL, genetic profiling has made possible the improvement in the diagnosis of aggressive forms. However, there are still scenarios that pose diagnostic difficulties. Genetic diagnosis will help to distinguish between primary and secondary cutaneous presentation, as well as between some pcBCLf and pcDLBCL forms.

## Figures and Tables

**Table 1:** Summary of genetic abnormalities reported in MF/SS [11,12,13,14,15,16,17,18,19,20,21,22,23,24,25,26,27,28].

Signaling	Mutations	CNVs (Deletions/Gains)	Druggable Pathway
Cell cycle	TP53 (19%), FAS (6%), RHOA (3%)	TP53 (83%), CDKN2A (40%), RB1 (39%), ATM (30%), CDKN1A (11%) MYC (35%)	Cell cycle regulators BET-inhibitors
JAK/STAT	STAT5B (4%), JAK3 (3%) JAK1 (1%), STAT3 (1%)	STAT3 (60%), STAT5B (60%), JAK2 (13%)	JAK/STAT inhibitors
MAPK	KRAS, NRAS, BRAF, MAP2K1, MAPK1 (<1–2%)	BRAF (18%)	MAPK inhibitors Tirosin-Kinase inhibitors
TCR/NF-κB	PLCγ1 (10%), CARD11 (5%), CCR4 (5%) CD28 (4%), PRKCQ (<1%), TNFRSF1B (2%)	TNFAIP3 (25%), NF-κB2 (25%) PRKCQ (30%), CARD11 (23%), TNFRSF1B (15%), PLCγ1 (5%)	Check-point inhibitors Calcineurine inhibitors NF-κB inhibitors PI3K inhibitors IKK inhibitors
Chromatin	POT1 (6%), DNMT3A (4%), TET2 (4%), KMT2C/KMT2D (3%), CREBBP (3%), NCOR1 (3%), BCOR (3%), TRRAP (5%), KDM6A (1%)	NCOR1 (80%), ARID1A (58%), DNMT3A (38%), ARID5B (29%), SETDB2 (28%), SMARCC1 (21%) TRRAP (10%)	HDAC inhibitors

**Table 2:** Proposal for an ampliseq panel of genes for mature T-cell malignant neoplasms [30].

Mature T-Cell Malignat Lymphoid Neoplasms: Candidate Genes for Amplification				
*TP53*	*FAS*	*MAP3K5*	*PLCG1*	*STAT5A*
*DNM3TA*	*GLI3*	*MAPK14*	*PTEN*	*NFATC2*
*BCOR*	*IDH2*	*NLRP2*	*RASA1*	*TET2*
*CARD11*	*IL6ST*	*NRAS*	*RB1*	*TNFRSF1B*
*CCR4*	*ARID1A*	*STAT3*	*PDCD1*	*JAK3*
*TRRAP*	*JAK1*	*RHOA*	*RELB*	*TRAF3*
*CD79A*	*CREBBP*	*KRAS*	*NFκ* *B1/2*	*TRAF6*
*SOCS1*	*JUNB*	*PIK3C2B*	*NF1*	*POT1*
*CTCF*	*NCOR1*	*KDM6A*	*SMARCB1*	*ZEB1*

**Table 3:** Differentially expressed microRNAs in CTCL.

miRNAprofile in CTCL Cases	Upregulated miRNAs	Downregulated miRNAs
CTCL global expression pattern	miR-155, miR-326, miR-663b, miR-711, miR-130b, miR-142-3p, miR-93-5p, miR-181a, miR-34a, miR-106b-5p, miR-148a-3p, miR-338-3p	miR-200b, and miR-203
Advanced MF	miR-155, miR-146a, miR-146b-5p, miR-342-3p, let-7i, miR-17~92 cluster, miR-106b~25, miR-106a~363 clusters, miR-181a/b, miR-21, miR-142-3p/5p	miR-200ab/429 cluster, miR-10b, miR-193b, miR-23b/27b, miR-203, miR-205, miR-141/200c
Sézary syndrome	miR-21, miR-214, miR-486	miR-23b, miR-31, miR-132
pcALCL	miR-155, miR-27b, miR-30c and miR-29b, miR-21, miR-142-3p/5p	miR-141/200c

**Table 4:** Selective/Personalized targeted approaches in MF/SS.

Signaling		Gene/Function	TargetedTherapy
TCR		PLCG1(+)	Calcineurine-inhibitors
		PTEN(−)	PI3K- inhibitors (duvelisib, idelalisib), mTOR- inhibitors
		RhoA(−)	Lenalidomide
		PRKG1(−)	MAPK- inhibitors
		PRKCQ(+)	MAPK-inh.
		CARD11(+)	Proteasome- inhibitors (bortezomib)
		TNFAIP3(−)	Proteasome- inhibitors
		NFKB2(+)	Proteasome- inhibitors
Membrane receptors/ Cytotoxicity or co-stimulation		CD30, CCR4, CD52, CD158	Brentuximab, Mogamulizumab, Alemtuzumab
		CD28(+)	Anti-CD80/CD86
		FusionCD28-CTLA4, CD28-ICOS(+)	Ipilimumab
		PD1/PDL1 (PDCD1(+/−)	PD1/PDL1 checkpoint- inhibitors
Cytokines regulation, metabolism, transcription, cell differentiation		TNFRSF1B(TNFR2)(+)	Protesome- inhibitors
		TNFRSF6(−)	Lenalidomide
		JAK1(+) JAK3(+) STAT3(+) STAT5B(+)	JAK- inhibitors (ruxolitinib)
		RFC-1, PARP	PARP- inhibitors
		Notch(+)	ϒ-secretase- inhibitors
Chromatin remodeling		ARID1A(−), ARID5B(−), SMARCC1(−), ARID1B, ARID4A, ARID2, ARID3A, SMARCA4, HD3(−)	HDAC- inhibitors
Transcription signaling		ZEB1(−)	MDM2- inhibitors
		IRF4(+)	Lenalidomide
		MYC(+)	BET- inhibitors
Histones methylation	Histones methyltranspherases	MLL2(+), MLL3(+), MLL4(+), SETD1A(−), SETD1B(−), SETDB2(−), SETD6(−), EZH2	ZH2- inhibitors (Tazemetostat)
	Histones demethylases	KDM6B(−)	HDAC- inhibitors
Histones acethylation	Histones acetiltranspherases	CREBBP(−)	HDAC- inhibitors
	Histones deacethylases	HDAC6(+)	HDAC- inhibitors (Vorinostat, romidepsine, resminostat, panobinostat, belinostat)
DNA methylation	DNA methyltranspherases	DNMT3A, DNMT3B	Hypomethylants (5-azacitidine, decitabine)
	DNA demethylation	TET1(−), TET2(−)	Protesome- inhibitors
Cell cycle		CDKN1B(−), CDKN2A(−)	CDK- inhibitors
		RB1(−), RPS6KA1(−), ATR(−)	Aurora kinase A- inhibitors
		TP53(−)	MDM2- inhibitors
miRNA (Oligonucleotide antagonist)		miR155(+)	Cobomarsen
Apoptosis (BH3 mimetic antagonist)		BCL2(+)	Venetoclax

**Table 5:** Summary of genetic abnormalities reported in cutaneous lymphomas other than MF/SS [41,42,43,44,45,46,47].

Other Cutaneous T-Cell Lymphomas	Gene/Translocation Target
Anaplastic Large cell lymphoma	t(2;5)(p23;q35)-----ALK/NPM (ALK+ systemic) 6p25.3 ----- DUSP22/IRF4 3q28-----TP63 NPM1-TYK2-----JAK/STAT IL6-JAK-STAT mutation DNMT3A, TP53 mutation
Breast Implant-Associated Anaplastic Large cell lymphoma	20q13.13 loss
Primary cutaneous γ/δ T-cell lymphoma	STAT5B mutation-----JAK/STAT SETD2 mutation
Subcutaneous Panniculitis-like T-cell lymphoma	HAVCR2 mutation-----TIM-3
Primary cutaneous aggressive epidermotropic CD8(+) T-cell lymphoma	CAPRIN1-JAK2-----JAK/STAT SELENOI-ABL1

**Table 6:** Summary of genetic abnormalities reported in primary cutaneous B-cell lymphomas.

Cutaneous B-Cell Lymphoma Subtype	Gene/Trasnslocation	Target/Gene
Marginal zone lymphoma (ccutaneous counterpart)	t(14;18)(q32;q21)	IgH/MALT
	t(3;14)(p14.1;q32)	FOXP1/IGH
	18q trisomy	FAS muations
Follicular lymphoma	t(14;18)(q32;q21)	IgH/BCL2 (rare in cutaneous counterpart)
	2p16.31 (amp REL)	
	14q32.32 del	
	1p36 del	
		TNFRS14 mutations
DLBCL, leg type	9p21	CDKN2A (or hypermethytation)
	3p.14.1	FOXP1
	6q del	BIMP1
	8q24	MYC
	3q27.3, 14q32	BCL6, IgH
		MYD88-mut, CD79B,
		CARD11,TNFAIP3/A20 (NF-κB)
	PDL1/PDL2-transl.	
	18q21.31–q21 ampl.	BCL2
